# Palmaris Longus in the Anubis Baboon (*Papio anubis*): A Conservative Single-Tendon Pattern with Rare Distal Bifurcation

**DOI:** 10.3390/biology15070562

**Published:** 2026-04-01

**Authors:** Ingrid C. Landfald, Rui Diogo, Kacper Ruzik, Judney Cley Cavalcante, Bento João Abreu, Magdalena Ciechanowska, Łukasz Olewnik

**Affiliations:** 1 Department of Clinical Anatomy, Mazovian Academy in Płock, 09-402 Płock, Poland; k.ruzik@mazowiecka.edu.pl (K.R.); m.ciechanowska@mazowiecka.edu.pl (M.C.); l.olewnik@mazowiecka.edu.pl (Ł.O.); 2 “VARIANTIS” Research Laboratory, Mazovian Academy in Płock, 09-402 Płock, Poland; judney.cavalcante@ufrn.br (J.C.C.); bento.abreu@ufrn.br (B.J.A.); 3 “VARIA” Research Laboratory, Mazovian Academy in Płock, 09-402 Płock, Poland; 4 Department of Anatomy, Howard University College of Medicine, Washington, DC 20059, USA; 5 Group of Study and Research in Human Anatomy (NEPAH), Department of Morphology, Federal University of Rio Grande do Norte, Natal 59078-970, RN, Brazil

**Keywords:** palmaris longus, *Papio anubis*, Cercopithecoidea, comparative primate anatomy, tendon morphology, anatomical variation, allometry

## Abstract

The palmaris longus (PL) remains insufficiently quantified in cercopithecoids (Cercopithecoidea), despite growing comparative data across primates. We examined adult archival material of the Anubis baboon (*Papio anubis*) to document PL presence, tendon configuration and topography, describe muscle–tendon morphometrics, and test for side-, sex- and size-related effects. A PL was present in all limbs. Two tendon configurations were observed: a single tendon inserting into the palmar aponeurosis (Type I, 87.0%) and a bifurcated tendon with both medial and lateral slips inserting into the palmar aponeurosis (Type II, 13.0%). No side- or sex-related differences were found in variant distribution. Males showed larger absolute values for selected measurements, but these differences were not independent of antebrachial size. PL lengths and interstyloid distances were strongly correlated with antebrachial size, indicating overall scaling with limb dimensions. These findings identify a species-level pattern in which PL is consistently present and predominantly unbranched, with only occasional distal bifurcation. The data establish a comparative anatomical baseline for Papio and broaden the available evidence on PL morphology in non-human primates.

## Simple Summary

The palmaris longus is a superficial antebrachial muscle whose tendon continues distally into the palmar aponeurosis and helps tension soft tissues during grasping. Although comparative descriptions exist across primates, species-level data remain limited for many cercopithecoids. In this study, we examined preserved thoracic limbs from adult olive baboons (*Papio anubis*) to describe the presence, structure, and dimensions of the palmaris longus muscle and its tendon. The muscle was present in all limbs and most often formed a single distal tendon, while a bifurcated configuration was uncommon. Overall, the findings indicate a predominantly conserved pattern in this species and provide a comparative anatomical baseline for further primate studies.

## 1. Introduction

The palmaris longus (PL) is a superficial flexor of the antebrachium that contributes to carpal flexion and to tensioning of the palmar fascia/aponeurosis. Across simiiform primates (Simiiformes), PL shows interspecific variation in presence, tendon architecture and distal insertion, indicating that this muscle should be documented at species level rather than inferred from a generalized primate pattern [1,2,3,4]. Existing comparative syntheses describe PL across major primate groups, but detailed morphometric datasets remain uneven, and cercopithecoids are still less comprehensively characterized than several other simiiform taxa [2,3,4].

The Anubis (olive) baboon, Papio anubis, was selected because it represents a well-defined papionin within Cercopithecinae with a locomotor repertoire centered on terrestrial quadrupedalism but retaining frequent grasping and manual manipulation during climbing, foraging and social behavior [5,6]. Accordingly, this species provides a useful species-level comparative datapoint within Cercopithecoidea, not as a surrogate for humans, but as a taxonomically and functionally distinct primate in which PL morphology has remained sparsely documented [1,4,7].

In this context, the principal biological question is whether PL in Papio anubis follows a tightly conserved gross-anatomical pattern or whether appreciable variation is already present within a single papionin species. Comparisons in the present manuscript are therefore used only to place the baboon findings within a broader primate framework, because equivalent species-level datasets remain limited for many non-human taxa [8,9,10,11,12].

Therefore, the aim of this study was to provide a species-level anatomical and morphometric characterisation of the PL in adult *Papio anubis*, with particular attention to tendon configuration and topography. Any classification of tendon patterns used herein is intended only as an operational reporting framework for this series, rather than as a general classification for primates.

## 2. Materials and Methods

### 2.1. Specimens

We examined 46 thoracic limbs from adult Anubis baboons *(Papio anubis),* originating from 13 males and 10 females, obtained post-mortem from zoological facilities and fixed in 10% neutral buffered formalin. The specimens were examined as archival, post-mortem material housed in the anatomical collection of the Department of Clinical Anatomy, Medical University of Lodz, Lodz, Poland. This study used exclusively archival, post-mortem material; no live animals were used and no procedures were performed. Accordingly, project authorisation under Directive 2010/63/EU and the corresponding Polish implementing act (Act of 15 January 2015 on the protection of animals used for scientific or educational purposes; Journal of Laws 2015, item 266, as amended) was not required for work on post-mortem archival specimens. Provenance is documented in collection records. Limb-level metadata included side (Left/Right; L = 23, R = 23).

### 2.2. Dissection

Standard antebrachial dissection exposed the PL muscle belly, the musculotendinous junction, and the tendon, with attention to the flexor retinaculum, palmar aponeurosis, and median nerve. The field of view included the proximal manus to document tendon topography and its relationship to the median nerve at the level of the carpus. The median nerve was exposed in the carpal region to document its relationship to the PL tendon; detailed tracing of muscular branches was not performed.

### 2.3. Variant Classification

For reporting purposes, PL tendon configuration was classified on gross dissection as Type I (a single, undivided tendon inserting into the palmar aponeurosis) or Type II (a macroscopic distal bifurcation into two discrete bands, both inserting into the palmar aponeurosis).

### 2.4. Measurements

Measurement definitions were developed a priori by the authors and implemented in a harmonised standard operating procedure (SOP), consistent with our previously published palmaris longus morphometric approaches [9,10].

Linear dimensions were obtained directly from the dissected specimens using digital calipers (resolution 0.01 mm). Distances were measured as straight-line segments between predefined bony landmarks. For each thoracic limb, we recorded:

(1) Antebrachial length (medial epicondyle → ulnar styloid (styloid process));
(2) PL muscle-belly length;
(3) PL tendinous length;
(4) Total PL length (PL_total = belly + tendon);
(5) Tendon width and thickness at the exit of the muscle belly (proximal);
(6) Mid-tendon width;
(7) Tendon width and thickness immediately proximal to the palmar aponeurosis (distal tendon).

In specimens where the tendon bifurcated, we additionally recorded:

(1) Lengths of both distal bands;
(2) Distance from the interstyloid line (between radial and ulnar styloids) to the point of bifurcation.

To account for limb size, selected variables were normalized to antebrachium length (unitless ratios). Each variable was measured twice by two investigators across two facilities; the mean of duplicate readings was used for analysis.

Tendon length was measured from the musculotendinous junction to the distal insertion into the palmar aponeurosis (Type I). In Type II specimens, tendon length was recorded from the musculotendinous junction to the bifurcation point, and the lengths of both distal bands were measured separately from the bifurcation point to their respective insertions. The interstyloid line was defined as the straight line between the radial and ulnar styloid processes; the interstyloid-to-bifurcation distance was measured from this line to the first level at which the two bands remained continuously distinct.

### 2.5. Photographic Documentation

Cadaveric photographs were acquired using a Sony α7 IV full-frame camera (Sony Corporation, Tokyo, Japan) equipped with a Sony FE 90 mm f/2.8 Macro G OSS (1:1) macro lens (Sony Corporation, Tokyo, Japan) mounted on a tripod and operated in manual mode under controlled lighting (~5600 K) against a neutral background. Images were captured to ensure comparable magnification and field of view between specimens, including the PL origin proximally and the palmar aponeurosis distally.

### 2.6. Statistical Analysis

Normality and homogeneity of variance were assessed with the Shapiro–Wilk and Levene tests, respectively. Depending on distributional assumptions, between-group comparisons (Type I vs. Type II, sex) used Student’s *t* tests (or Welch’s *t* when variances were unequal) or the Mann–Whitney U test. Because both thoracic limbs were available for each individual, left–right comparisons were performed within individuals using paired tests. For between-group analyses (Type I vs. Type II; sex), we addressed within-individual dependence by using individual-level summaries (mean of left and right limbs per subject) in sensitivity analyses; conclusions were unchanged relative to limb-level analyses. Where relevant, the unit of analysis is specified (limb-level for descriptive morphometrics; individual-level for sensitivity testing). Proportions (e.g., presence of a distal split) were compared with Fisher’s exact test. Statistical significance was set at α = 0.05 (two-sided). Selected variables were additionally analysed after normalisation to antebrachium length. All analyses were performed in Python (v3.14.3, Python Software Foundation, Wilmington, DE, USA) using the pandas and SciPy libraries.

## 3. Results

### 3.1. Prevalence and Gross Architecture

A PL was present in all 46 thoracic limbs; no unilateral or bilateral agenesis was identified. The PL coursed superficially along the caudal aspect of the distal antebrachium towards the carpus without aberrant paths.

### 3.2. Gross Anatomy and Topography

**Figure 1:**
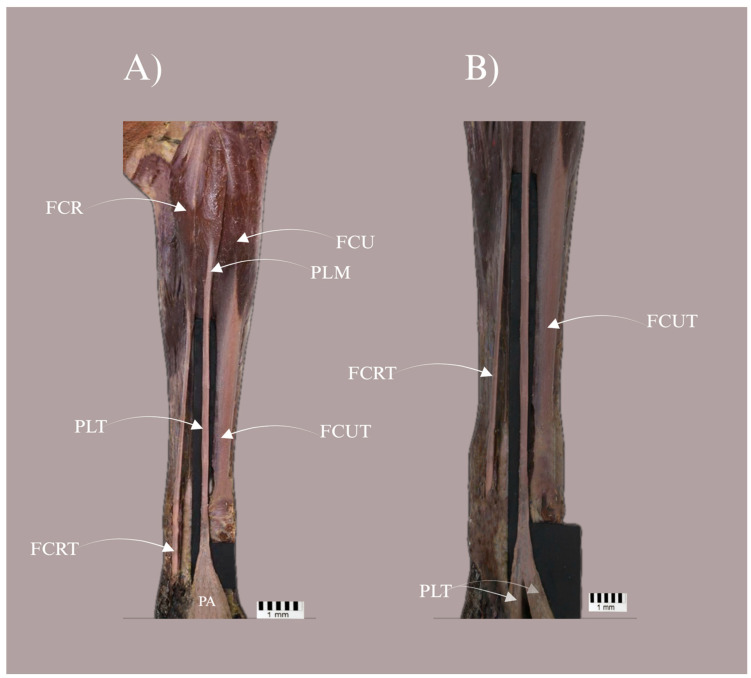
Distal architecture of the palmaris longus in *Papio anubis*. (**A**) Type I configuration showing a single palmaris longus tendon (PLT) continuing from the palmaris longus muscle (PLM) and inserting into the palmar aponeurosis (PA). (**B**) Type II configuration demonstrating a bifurcation of the PLT near the insertion, with both medial and lateral bands inserting into the palmar aponeurosis (PA). **Abbreviations:** FCR, flexor carpi radialis; FCU, flexor carpi ulnaris; FCRT, tendon of flexor carpi radialis; FCUT, tendon of flexor carpi ulnaris; PLM, palmaris longus muscle; PLT, palmaris longus tendon; PA, palmar aponeurosis.

**Figure 2:**
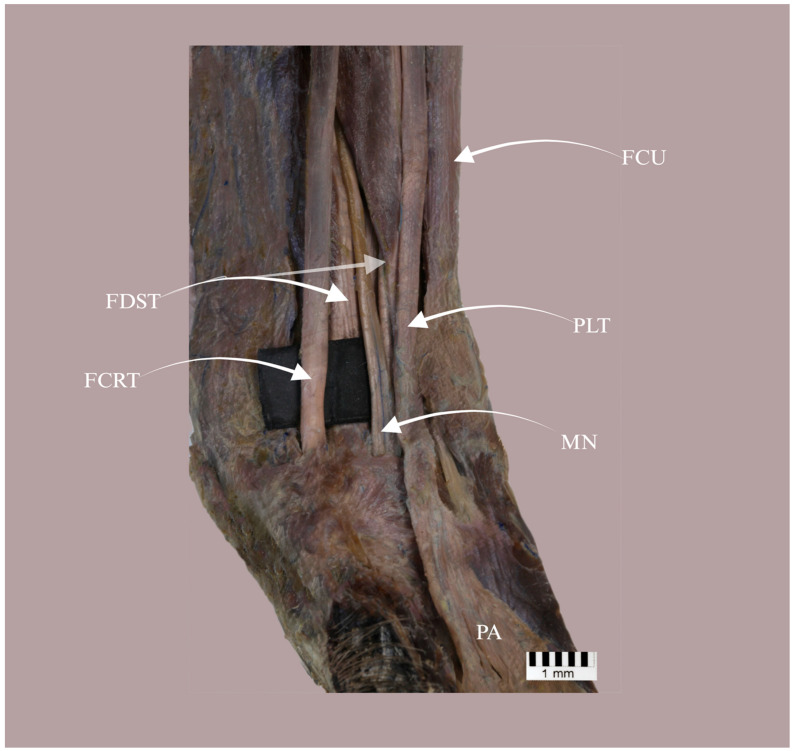
Palmar view of the distal antebrachium and carpal region in *Papio Anubis* demonstrating the topographic relationship between the palmaris longus tendon (PLT) and the median nerve (MN). The PLT courses superficially along the midline between the tendon of flexor carpi radialis (FCRT) and flexor carpi ulnaris (FCU) toward the palmar aponeurosis (PA), whereas the MN lies deep to the PLT at the level of the carpus. FDST, flexor digitorum superficialis tendons. Scale bar = 1 mm.

In all specimens, the PL arose from the common flexor origin at the medial epicondyle of the humerus, with the proximal attachment composed predominantly of short fleshy fibres; these fibres blended secondarily with the adjacent antebrachial fascia rather than taking origin independently from the fascia. On gross inspection, the belly was narrow and elongated, tapering into a tendon in the distal antebrachium. At the level of the carpus, the PL tendon remained superficial to the flexor retinaculum and continued distally towards the palmar aponeurosis (Figure 1). The median nerve was identified in the carpal region and consistently lay deep to the PL tendon (Figure 2); however, the nerve was not traced proximally to the muscle belly and intramuscular motor entry points or branching patterns were not assessed. In Type II specimens, bifurcation occurred within the proximal manus at a mean distance of 30.78 mm distal to the interstyloid line (Table 3), forming two discrete slips (medial and lateral) inserting separately into the palmar aponeurosis.

### 3.3. Variant Types

To standardise reporting, PL tendon configuration was classified on gross dissection as Type I (a single, undivided tendon inserting into the palmar aponeurosis) or Type II (a macroscopic bifurcation near the insertion into two discrete bands, both inserting into the palmar aponeurosis). A “bifurcation” was recorded only when the two bands were clearly separable over a measurable length. The bifurcation point was defined as the first level at which the bands became continuously distinct; its position was quantified as the distance from the interstyloid line to the bifurcation point. Band lengths were measured from the bifurcation point to their insertion into the palmar aponeurosis.

Using these operational definitions, two configurations were observed. Type I predominated (40/46, 87%), whereas Type II occurred in 6/46 limbs (13%). By-type descriptive morphometrics are summarised in Table 1. In Type II specimens, the bifurcation point was located within the proximal manus; the interstyloid-to-bifurcation distance is summarised in Table 2.

(1) Type I featured a single tendon inserting into the palmar aponeurosis—Figure 1A.
(2) Type II showed a bifurcation near the insertion, forming two discrete bands that both inserted into the palmar aponeurosis—Figure 1B; presence of both bands and quantitative band metrics are summarised in Table 3.

### 3.4. Between-Type Comparisons of Tendon Geometry

At the exit of the muscle belly (proximal), the tendon was wider and thicker in Type I (*p* < 0.001 for both width and thickness). At the distal end, width was numerically larger in Type I but did not reach significance (*p* = 0.051), and distal thickness did not differ significantly.

### 3.5. Length Measures and Size Normalisation

Raw muscle-belly, tendinous, and total PL lengths tended to be greater in Type I, but these measures scaled with antebrachium size. After normalising to antebrachium length, no differences remained between types (all *p* > 0.45), indicating that the apparent raw-length contrasts primarily reflect overall limb size.

## 4. Discussion

Taken together, our series characterises the PL in *Papio anubis* as a strongly conserved species-level pattern, with universal presence, predominance of a single tendon inserting into the palmar aponeurosis, and an uncommon but recurrent distal bifurcation near the insertion. The central contribution of the study is therefore descriptive and comparative within *Papio anubis* itself: it establishes the gross topography, frequency of tendon configurations and size-related morphometric context for a papionin taxon for which detailed PL data have been limited.

Within Cercopithecoidea, anatomical syntheses generally describe a well-developed PL with a relatively standard insertion pattern, and the present findings are consistent with that broader cercopithecoid background [1,3,4,7]. In this respect, *Papio anubis* does not appear to show the degree of gross-anatomical lability often emphasized in human-focused literature, but rather a comparatively constrained tendon pattern at the adult stage. Our data therefore support the view that the baboon PL should first be interpreted as a conserved cercopithecoid structure, before any broader extrapolation is attempted.

From a functional standpoint, the PL contributes to carpal flexion and to tensioning of the palmar aponeurosis, thereby stabilising palmar soft tissues during grasp [13]. In *Papio anubis*, which combines terrestrial quadrupedal locomotion with climbing and frequent manual grasping/manipulation, the consistent presence of the PL and the preserved distal relationship between its tendon and the median nerve suggest a stable superficial flexor arrangement compatible with repeated loading of the antebrachium and manus. The rare bifurcation observed in Type II may modestly redistribute tension within the palmar aponeurosis, but any functional significance remains hypothetical and requires biomechanical testing.

At a broader primate level, published dissections indicate that the degree of constraint on PL presence and morphology differs among clades. Cercopithecoids, and especially papionins and macaques, are generally described as retaining a well-developed PL with comparatively limited reports of agenesis [1,3,4,7], whereas variability appears more frequently in parts of Hominoidea [3,4,11,12,14]. This broader comparison is relevant here not because Papio anubis is being treated as a direct model of the human condition, but because it helps position the baboon findings within a primate spectrum extending from relatively conserved to more variable PL morphology.

Published datasets provide descriptive context for interspecific differences in PL tendon–belly proportions, but direct comparisons based on raw lengths should be interpreted cautiously because body size, limb proportions and measurement protocols vary among taxa [2,14,15,16].

Within that limited comparative framework, human data simply illustrate one end of the primate spectrum rather than a default reference state. Reported adult series show substantial variation in PL presence and insertion, with pooled absence rates approaching 20% in some analyses [10,11,12,17]. The contrast with the universally present and morphologically constrained PL observed in Papio anubis therefore supports a comparative, not translational, inference: high variability should not be assumed across primates, and species-level datasets remain essential.

A similar logic applies to developmental interpretation. Human fetal series indicate that PL variability can already be established prenatally, with multiple insertional morphotypes and cases of unilateral or bilateral absence [9]. These data are informative as a developmental reference, but they cannot be projected directly onto *Papio*. Because our material comprises adults only, the present study cannot determine whether transient prenatal variants occur in *Papio* and are later remodelled, or whether developmental outcomes are constrained earlier in cercopithecoids. The current dataset therefore provides a baseline for future ontogenetic work rather than evidence of shared developmental trajectories.

This study has several limitations. First, it is restricted to a single species (*Papio anubis*), and broader sampling across cercopithecoids, particularly additional papionins, will be required to establish how widely the bifurcation pattern occurs and whether its topography is consistent across related taxa. Second, all specimens were derived from zoo-maintained adults, which may differ in habitual loading and activity patterns from wild populations; however, the highly stereotyped morphology observed suggests that such environmental variation is unlikely to alter the core anatomical pattern. Third, the absence of ontogenetic material (fetal, juvenile) precludes direct evaluation of developmental trajectories and the potential presence of transient variants prior to maturity. Although the median nerve was identified in the carpal region to document its relationship to the PL tendon, we did not trace the nerve proximally to the muscle belly or assess intramuscular branching; therefore, no conclusions regarding PL innervation can be drawn from this series.

Future work should prioritise (1) expanded taxonomic sampling within cercopithecoids to map the frequency and topography of tendon-configuration variants, including whether bifurcation patterns comparable to Type II occur in other papionins or macaques; (2) systematic documentation of PL innervation in *Papio* across larger, bilaterally sampled series to assess the prevalence of non-median supply suggested by single-case reports [18]; and (3) ontogenetic series in cercopithecoids, where available, to test whether prenatal variants arise and are later normalised, as reported in humans [9].

Overall, the PL in *Papio anubis* is best interpreted as a conserved adult cercopithecoid pattern documented at species level. Its value lies in refining comparative primate anatomy: first by clarifying the morphology of an understudied papionin taxon, and second by providing a grounded reference against which broader evolutionary or developmental hypotheses can later be tested.

## 5. Conclusions

In adult *Papio anubis*, the palmaris longus was present in all examined limbs and showed a predominantly single-tendon insertion into the palmar aponeurosis, with a rare but stereotyped distal bifurcation. These findings establish a species-level anatomical baseline for a papionin cercopithecid and support cautious comparative use of the baboon data within broader primate morphology, without assuming direct equivalence to the human pattern.

## Figures and Tables

**Table 1:** Descriptive statistics by type (mean ± SD).

Variable	Type I	Type II
Antebrachium length (mm)	264.25 ± 4.99	262.95 ± 2.80
Muscle belly length (mm)	92.82 ± 3.94	91.80 ± 2.65
Tendon length (mm)	163.40 ± 5.63	162.32 ± 4.71
Total PL length (mm)	256.22 ± 9.44	254.12 ± 7.26
Mid-tendon width (mm)	2.91 ± 0.19	2.36 ± 0.11
Tendon width (mm)	23.49 ± 2.48	21.00 ± 1.70
Tendon thickness (mm)	2.70 ± 0.22	2.68 ± 0.22
Tendon thickness at belly exit (mm)	2.38 ± 0.22	2.09 ± 0.10
Interstyloid-to-bifurcation distance (mm)	—	30.78 ± 0.84
Lateral band length (mm)	—	32.32 ± 4.85
Medial band length (mm)	—	26.31 ± 3.17
Tendon width at belly exit (mm)	2.11 ± 0.18	1.93 ± 0.07

Variables are reported per limb. Units are millimetres (mm).

**Table 2:** Distal bands—presence by type (medial and lateral bands inserting into the palmar aponeurosis).

Type	Medial Band Present (n, %)	Lateral Band Present (n, %)
I	0 (0.0%)	0 (0.0%)
II	6 (100.0%)	6 (100.0%)

**Table 3:** Band metrics (Type II only).

Measure	Mean	SD	Median	Min–Max
Medial band length (mm)	26.31	3.17	27.16	22.33–29.42
Lateral band length (mm)	32.32	4.85	35.13	25.72–36.27
Interstyloid-to-bifurcation (mm)	30.78	0.84	30.69	29.94–31.92

## Data Availability

The data supporting the findings of this study, including raw morphometric measurements and derived indices, are available from the corresponding author upon reasonable request.
